# Smartphone-Based Dopamine Detection by Fluorescent Supramolecular Sensor

**DOI:** 10.3390/molecules27217503

**Published:** 2022-11-03

**Authors:** Rossella Santonocito, Nunzio Tuccitto, Andrea Pappalardo, Giuseppe Trusso Sfrazzetto

**Affiliations:** 1 Department of Chemical Sciences, University of Catania, Viale A. Doria 6, 95100 Catania, Italy; 2 Laboratory for Molecular Surfaces and Nanotechnology—CSGI, 95125 Catania, Italy; 3 National Interuniversity Consortium for Materials Science and Technology (I.N.S.T.M.) Research Unit of Catania, 95125 Catania, Italy

**Keywords:** cavitand, dopamine, supramolecular, smartphone, saliva

## Abstract

Supramolecular recognition of dopamine by two quinoxaline cavitands was studied in solution by fluorescence titrations, ESI-MS and ROESY measurements. In addition, the tetraquinoxaline cavitand was dropped onto a siloxane-based polymeric solid support, obtaining a sensor able to detect dopamine in a linear range of concentrations 10 Mm–100 pM, with a detection limit of 1 pM, much lower than the normal concentration values in the common human fluids (plasma, urine and saliva), by using a simple smartphone as detector. This sensor shows also good selectivity for dopamine respect to the other common analytes contained in a saliva sample and can be reused after acid–base cycles, paving the way for the realization of real practical sensor for human dopamine detection.

## 1. Introduction

Dopamine (DA), a phenethylamine derivative produced by the adrenal medulla, is fundamental in many brain functions. In particular, alteration of DA levels can be related to different diseases, such as Alzheimer [1], schizophrenia [2], Parkinson, Huntington [3] attention-deficit hyperactivity disorder [4] and paragangliomas [5]. For these reasons, DA sensing is crucial to monitoring the human health condition. In normal conditions, the DA concentration in plasma is around 20 ng/mL [6], 18.9 pg/mL in saliva [7] and is 0.2–1 mg/mL in human urine [8]. Different DA detection methods have been reported, including chromatographic [9], electrophoretic [10], electrochemical [11,12,13] and methods based on Surface Plasmon Resonance [14,15]. However, these techniques require expert personal, high-cost, long and complicated analysis. The possibility to obtain a *point-of-care* DA detection method is undoubtedly interesting and useful for a rapid screening of the DA levels in blood, saliva and urine. To this end, optical or colorimetric sensors are more useful [16,17,18,19,20,21,22,23,24,25,26,27,28]. In addition, the use of a simple smartphone as detector leads to the possibility to a “homemade” DA levels detection. Few examples of smartphone-based methods able to determine the DA concentration, in micromolar ranges, have been reported [29,30,31,32,33,34,35]. In this context, the possibility to restore the starting sensor leads to the opportunity to reuse the device. This goal can be achieved by exploiting the principle of supramolecular chemistry, and in particular the non-covalent interactions between a receptor/host (sensor) and the analyte/guest (DA). To the best of our knowledge, only a few examples of non-covalent sensing of DA have been reported, also exploiting cyclodextrins [36,37,38,39], calixarenes [40,41], pillararenes [42,43] and cucurbiturils [44,45,46,47] in these cases with micromolar ranges of linearity and limits of detection.

Here, we report the first example of DA detection by a reusable solid device exploiting a quinoxaline cavitand as supramolecular receptor, by using a smartphone as detector. This sensor shows, on siloxane-based polymeric solid state, good linearity range (10 mM–100 pM), low limit of detection value (1 pM, corresponding to 87 fg of DA) and excellent selectivity for DA respect to the other common analytes contained in the saliva. Notably, the picomolar limit of detection allows the detection of DA concentrations in real saliva samples. The novelty of this proposed sensor is the possibility to detect DA by supramolecular approach, obtaining a reusable device able to be used with a smartphone as detector. The possibility to monitor DA levels by a simple smartphone leads to the easy homemade DA monitoring. In addition, the high resolution of modern cameras allows the detection of slight changes of color/emission, not easily detectable by the naked eye.

**Chart 1:**
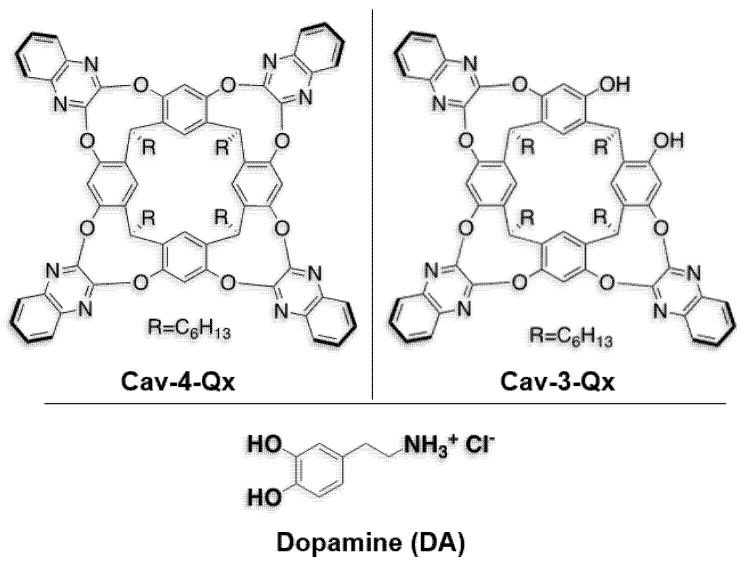
Chemical structures of quinoxaline cavitands and dopamine hydrochloride (DA) used in this work.

To choose the optimal receptor for dopamine, we focused our attention on two quinoxaline cavitands, **Cav-4-Qx and Cav-3-Qx**, reported in Chart 1, containing four and three quinoxaline walls, respectively. In particular, DA shows a molecular volume of 315 Å^3^ (considering van der Waals radius). Considering the ideal guest/host volume ratio (~0.55, known as “The 55% solution”) suggested by Rebek and Mecozzi [48,49], quinoxaline cavitands shows the perfect inner volume (580 Å^3^) to include DA inside the quinoxaline cavity.

## 2. Results

**Scheme 1:**
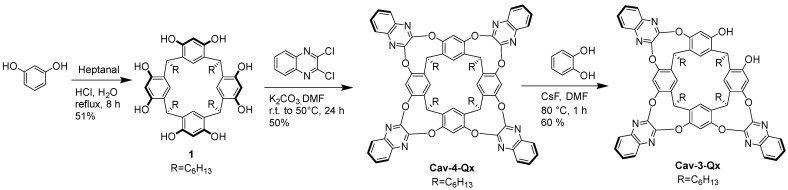
Synthesis of **Cav-4-Qx** and **Cav-3-Qx**.

**Cav-4-Qx** and **Cav-3-Qx** have been synthesized following the reactions showed in Scheme 1. In particular, 1,3 dihydroxybenzene and heptanal have been mixed in acid solution, in high concentration value, leading to resorcinarene **1**, which in the presence of potasium carbonate and an excess of 2,3 dichloroquinoxaline, leads to **Cav-4-Qx**. Excision reaction, using CsF as base and catechol, allows to remove selectively one quinoxaline unit, leading to **Cav-3-Qx**.

**Figure 1:**
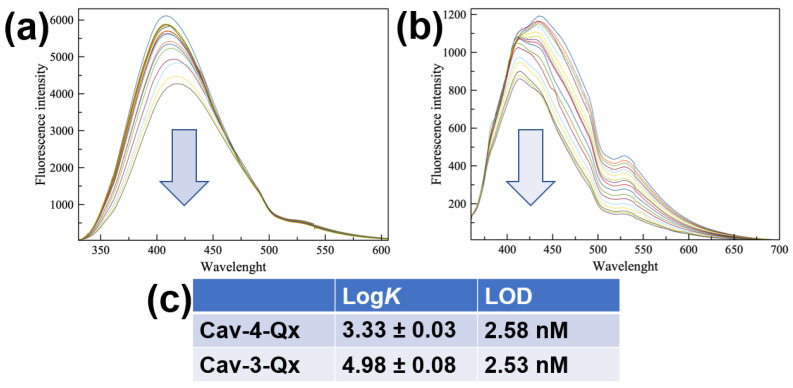
Fluorescence titrations between DA and **Cav-4-Qx** (**a**) and **Cav-3-Qx** (**b**) in CHCl_3_ ((Host) = 1 × 10^−5^ M, (DA) = 0~1.13 × 10^−4^ M, λ_ex_ 320 and 340 nm for **Cav-4-Qx** and **Cav-3-Qx**, respectively); (**c**) binding constant values of supramolecular complexes with DA calculated by HypSpec version 1.1.33 [54,55,56,57], detection limit was calculated by the method of the calibration curve, using the formula LOD = 3σ/K, where s is the standard deviation of the blank and K is the slope of the calibration curve.

Due to the easy oxidation of dopamine in air, sensing properties have been evaluated by using dopamine hydrochloride (DA, see Chart 1), more stable in normal conditions. Figure 1a,b show the fluorescence titrations of **Cav-4-Qx** and **Cav-3-Qx**, respectively, with DA in chloroform solution (see the Supplementary Materials). **Cav-4-Qx** and **Cav-3-Qx** have been excited at 320 nm and 340 nm, respectively. 

In both cases, a quenching of cavitand emissions can be observed, due to a PET mechanism between quinoxaline units of hosts and DA, also observed with other organic guests in solution [50,51,52,53]. Emission maximum at 408 nm for **Cav-4-Qx** and 436 nm for **Cav-3-Qx** have been used to calculate the binding affinity for DA, by using HypSpec software.

In particular, **Cav-4-Qx** undergoes to a slight redshift of emission upon the addition of DA (10 nm), probably due to the decrease in energy of the excited state in the supramolecular complex. **Cav-3-Qx** shows two emission bands (at 411 and 435 nm), which decrease differently. These changes of emission can be related to the variation of energy levels involved in the fluorescence emission after the inclusion of DA.

Figure 1c shows the binding constant values, assuming a 1:1 stoichiometry, as suggested by Job’s Plot experiments (see the Supplementary Materials), and the relative limit of detections (LOD) calculated by the method of the calibration curve. In particular, we observed that **Cav-3-Qx** shows an affinity for DA more than one order of magnitude higher than **Cav-4-Qx**, probably due to the absence of a quinoxaline wall (thus leading to a less steric hindrance) and the presence of two free phenolic groups (leading to the possibility to establish hydrogen bonds with the guest). The formation of the supramolecular complexes was further supported by ESI-MS and ROESY measurements. In particular, ESI-MS analysis of an equimolar solution of **Cav-4-Qx** and DA shows a peak at *m/z* 1482.9, relative to the 1:1 supramolecular complex (see the Supplementary Materials). The same result has been obtained by the ESI-MS of an equimolar solution of **Cav-3-Qx** and DA, in which a peak at *m/z* 1355.6 supported the formation of the 1:1 supramolecular complex (see the Supplementary Materials).

**Figure 2:**
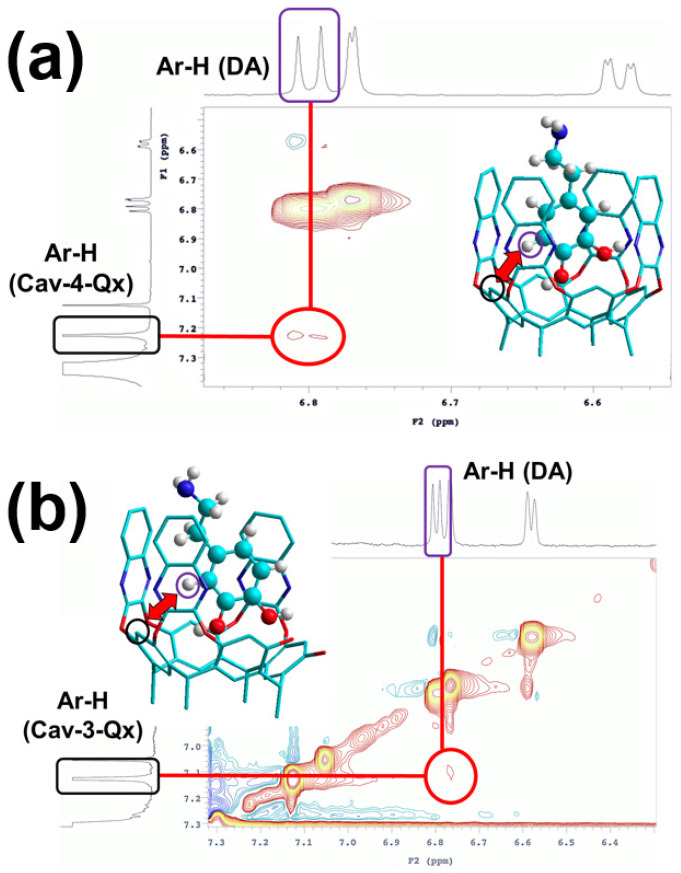
Detail of ROESY spectra of (**a**) **Cav-4-Qx** and DA and (**b**) **Cav-3-Qx** and DA in CDCl_3_/CD_3_OD (6/1) ((Host) = (DA) = 1 × 10^−3^ M); insets show the minimized structure (force field MM+) of the supramolecular complexes, highlighting the functional groups involved in the ROE contacts (host signals are shown in black, DA is highlighted in violet, hydrogens of the hosts are omitted for clarity).

Recognition of DA by **Cav-4-Qx** and **Cav-3-Qx** was further supported by the NMR ROESY experiments. In particular, ROESY spectrum of equimolar CDCl_3_/CD_3_OD (6/1) solution of **Cav-4-Qx** and DA shows ROE contact between the upper-rim aromatic proton of resorcinarene scaffold and the aromatic proton of DA (Figure 2a). Similarly, ROESY spectrum of the supramolecular complex between **Cav-3-Qx** and DA shows similar ROE contact (Figure 2b). In both cases, the ROESY experiment suggests the inclusion of the aromatic ring of DA inside the quinoxaline cavities (^1^H NMR signals of hosts and guest did not undergo to a significant chemical shift change). These supramolecular complexes can be stabilized by CH-π interactions between aromatic protons of DA and quinoxaline walls of the receptors. In addition, **Cav-3-Qx** can also further stabilize DA by hydrogen bonds between free phenolic OH groups.

In order to obtain a real practical sensor able to detect DA in real samples, we realized a test strip dropping **Cav-4-Qx** and **Cav-3-Qx** onto a siloxane-based polymeric substrate (see Materials and Methods for the details). Unfortunately, **Cav-3-Qx** dropped onto the siloxane-based polymeric support did not show a significant emission of fluorescence; thus, our studies were performed with **Cav-4-Qx.** The presence of free OH groups in **Cav-3-Qx** likely leads to a quenching of emission after deposition. This phenomenon has also been detected using silica gel as solid substrate, during the TLC analyses.

Test strip was prepared by dropping 2 μL of **Cav-4-Qx** (1 mM in CHCl_3_) onto the polymeric support. The as-prepared solid sensor was illuminated by UV lamp at 365 nm in a dark chamber room and emission image was acquired by a commercial smartphone (see Materials and Methods for the details). The use of a smartphone as a detector is, in this case, fundamental due to the small emission changes of the **Cav-4-Qx** upon the exposure to few amounts of DA (femtograms of compound). At this stage, the naked eye cannot recognize this difference of emission.

**Figure 3:**
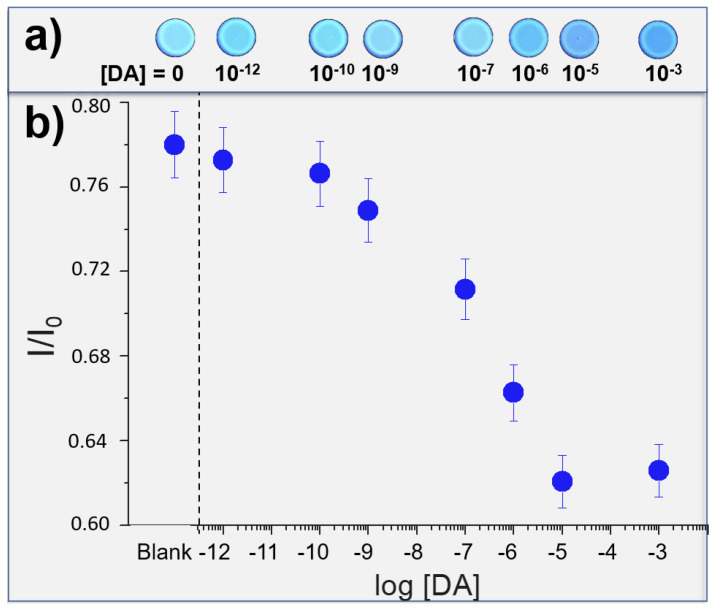
(**a**) photos of test strips exposed to increased amounts of DA; (**b**) correlation between the log[DA] and the normalized emission values of the solid sensor (I/I_0_, where I and I_0_ are the Gray channel emission values of **Cav-4-Qx** and control probe, respectively).

Then, DA solutions (from 10^−3^ M to 10^−12^ M solution in EtOH) were nebulized on to the solid sensor and, after the solvent evaporation, new images were acquired (see Figure 3a). These images were elaborated by Fiji software [58], in particular, each image has been converted into RGB channel values, and then converted into Gray scale value (G) by using the formula G = (R_value_ + G_value_ + B_value_)/3, thus obtaining a single value for each pixel. The emission intensities of G relative to the probe have been compared with the control molecule (phenanthrene, a fluorescent molecule that does not interact with the DA), and these normalized values (ratio between the intensities of the probe and the control) have been reported in Figure 3b, compared with the emission of the **Cav-4-Qx** without DA (Blank). We can observe detection properties from 1 mM to 1 pM (detection limit, LOD), corresponding to an amount of DA nebulized of 87 mg and 87 fg, respectively (these amounts have been calculated considering the concentration of the solution nebulized and represent the total amount of DA nebulized in each experiment). In addition, a linear trend can be found in the range 10 μM–100 pM. Considering that, DA concentrations in the common human fluids are 0.13 μM, 7 μM and 0.124 nM in plasma, urine and saliva, respectively, our solid sensor should be employed to detect DA in these human matrixes.

We supported in solution the formation of a supramolecular complex between **Cav-4-Qx** and DA by ROESY and ESI-MS experiments; however, the formation of a similar complex also on solid state cannot be demonstrated.

**Figure 4:**
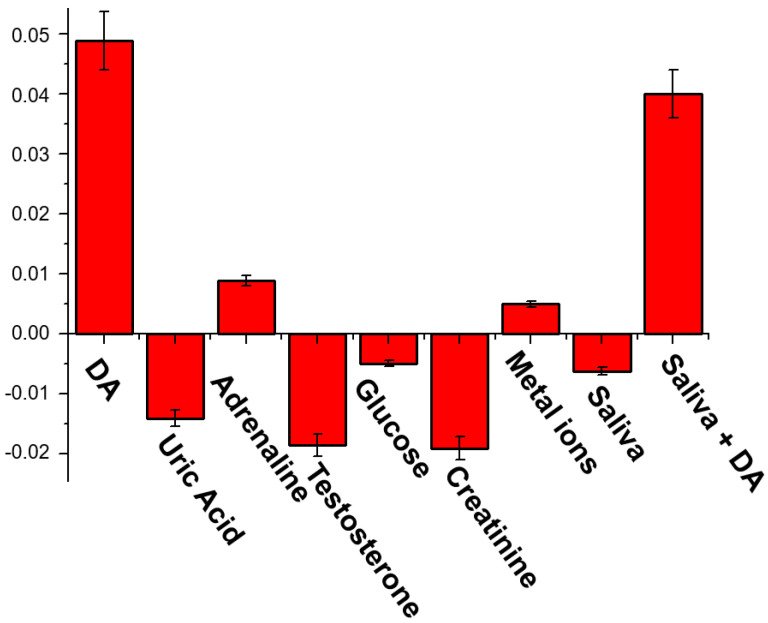
Selectivity tests: emission response (I-I_0_, where I and I_0_ are the Gray channel emission values of each test image after and before the analyte exposition, respectively) of the solid sensor to different analytes dissolved in artificial saliva (composition: NaCl 2.9 mg/mL, KCl 6.35 mg/mL, Na_2_HPO_4_ 1.7 mg/mL, KH_2_PO_4_ 1.65 mg/mL, KSCN 0.8 mg/mL, CaCl_2_ × 2H_2_O 0.85 mg/mL, Urea 1 mg/mL, NH_4_Cl 0.8 mg/mL, ascorbic acid 0.01 mg/mL), metal ions (MgCl_2_ 1 mg/mL, MnCl_2_ 1 mg/mL, FeCl_2_ 1 mg/mL, CuCl_2_ 1 mg/mL, ZnCl_2_ 1 mg/mL), real saliva and real saliva containing 0.1 nM of DA.

To validate the possibility to use our solid sensor in real life and demonstrate the selectivity for DA with respect to the other common analytes contained in the human fluids, we chose to use saliva as real sample, due to the ease and stress-free sampling and the lower DA concentration respect to the plasma and urine. Firstly, we tested the response of our sensor to some important analytes contained in the human saliva (Figure 4) [59]. In particular, we exposed test strip to DA (0.1 nM), uric acid (20 μM), adrenaline (0.1 nM), testosterone (0.1 pM), glucose (72 μM), creatinine (1 μM) and some metal cations (Mg^2+^, Ca^2+^, Mn^2+^, Fe^2+^, Cu^2+^, Zn^2+^), dissolved in artificial saliva [60]. DA and adrenaline led to an enhancement of the emission, significant only in the case of DA. The other analytes leaded to a quenching of the strip emission. 

Then, we exposed our test strip to a real saliva sample, also observing in this case a decrease of the emission of the cavitand onto the solid support. The same sample, after the addition of 0.1 nM of DA, generated an increase of the emission, comparable to the initial 0.1 nM standard DA solution, suggesting the possibility to use this prototype in real samples.

**Figure 5:**
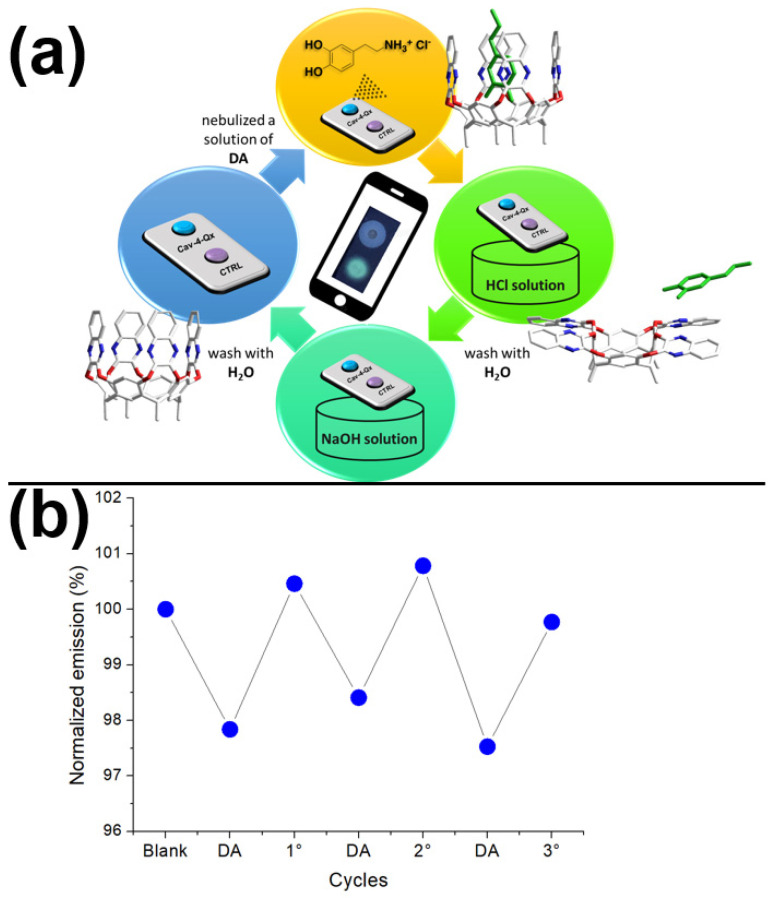
Recovery test: (**a**) representation of acid–base cycle and switch of the **Cav-4-Qx** vase-kite conformations (DA is reported in green); (**b**) normalized emission response (respect to the emission of the blank) of the solid sensor without (blank), after nebulization of DA (10^−3^ M in EtOH) and after acid–base cycles.

One of the main goals of the Supramolecular approach in the sensing is the reversibility of the non-covalent interactions, leading to the possibility to restore the starting sensor [54,55,57,60]. In this context, we exploited the ability of the quinoxaline cavitand to release a guest inside the hydrophobic cavity by switching from the vase to the kite conformation, at low pH values [61]. Due to the reversibility of this equilibrium, the further increase of the pH value leads to the restoration of the vase conformation, recovering the starting recognition properties (see Figure 5a). Figure 5b shows the possibility to recover the solid sensor by acid–base cycles (see Materials and Methods for details). In particular, after the first DA exposure that leads to a quenching of the emission, the solid sensor can be restored by immersion into a HCl solution (1 mM in water), followed by a second immersion into a NaOH solution (1 mM in water). After this treatment, the emission of the **Cav-4-Qx** is almost restored. This behavior has been demonstrated for three cycles (see Figure 5b).

Other sensoristic systems has been used by employing a smartphone as detector for DA sensing in real samples. In particular, DA has been detected in urine, with a linear response in the range of micromolar, and a limit of detection under micromolar concentration [8,32]; in plasma with a linear response in the range of nanomolar—micromolar and a detection limit of micromolar concentration [34]; and in sweat with a linear response up to millimolar concentration and a nanomolar limit of detection [31]. Our device, with a picomolar limit of detection and a linearity in the range 10 pM–10 μM, shows better results in these analytical parameters.

## 3. Materials and Methods

### 3.1. General Experimental Methods

The NMR experiments were carried out at 27 °C on a Varian UNITY Inova 500 MHz spectrometer (^1^H at 499.88 MHz, Varian-Agilent, Santa Clara, CA, USA) equipped with pulse field gradient module (Z axis) and a tunable 5 mm Varian inverse detection probe (ID-PFG). ESI mass spectra (AB Sciex, Milan, Italy) were acquired on an API 2000 using CH_3_CN (positive ion mode). Luminescence measurements were carried out using a Cary Eclipse Fluorescence spectrophotometer (Agilent, Santa Clara, CA, USA) with resolution of 0.5 nm, at room temperature. The emission was recorded at 90° with respect to the exciting line beam using 5:5 slit widths for all measurements. All chemicals were reagent grade and were used without further purification. 3D minimized structures reported in the manuscript and molecular volume of DA and cavitands were obtained using HyperChem v8.0.7, MM+ force field.

### 3.2. Synthesis of **1**

A total of 16.5 g (150 mmol) of resorcinol was dissolved in 20 mL of ethanol and 20 mL of HCl conc. The reaction temperature was cooled at 5 °C and 17.1 mL (150 mmol) of heptanal were added slowly in one hour. The temperature was further increased to reflux for 8 h obtaining a solid precipitate. After the addition of water, the precipitate was filtered and washed with water. Compound **1** (15.8 g, 51%) was crystallized by methanol. ^1^H NMR (acetone-*d_6_*, 500 MHz) δ 0.88 (t, *J* = 6.5 Hz, 12H, CH_3_), 1.29 (bs, 32H, CH_2_), 2.28 (q, *J* = 6.5 Hz, 8H, CHCH_2_), 4.23 (t, *J* = 8 Hz, 4H, CH), 6.23 (s, 4H, Ar–H), 7.54 (s, 4H, ArH), 8.50 (s, 8H, OH). ESI-MS *m/z* 825 [M+H]^+^. Anal. Calcd for C_52_H_72_O_8_: C, 75.69; H, 8.80. Found: C, 75.62; H, 8.71. 

### 3.3. Synthesis of **Cav-4-Qx**

A total of 1.40 g (1.65 mmol) of 1, 2.00 g (10.0 mmol) of 2,3-dichloroquinoxaline and 1.50 g (10.0 mmol) of anhydrous potassium carbonate were mixed in 30 mL of dry DMF. The reaction was stirred at room temperature for 8 h, and then heated at 50 °C for 18 h. The reaction was quenched with 60 mL of water and filtered to obtain a crude solid. **Cav-4-Qx** (1.10 g, 50%) was isolated after column chromatography (CHCl_3_:EtOAc 95:5). 1H NMR (CDCl_3_, 500 MHz) δ 0.92 (t, *J* = 6.5 Hz, 12H, CH_3_), 1.29 (bs, 32H, CH_2_), 2.28 (brq, *J* = 6.5 Hz, 8H, CHCH_2_), 5.54 (t, *J* = 8.0 Hz, 4H, CH), 7.21 (s, 4H, ArH), 7.47–7.79 (m, 16H, ArH), 8.15 (s, 4H, ArH). ESI-MS m/z 1329 [M+H]^+^. Anal. Calcd for C_84_H_80_N_8_O_8_: C, 75.88; H, 6.06; N, 8.43; O, 9.63. Found C, 75.81; H, 6.03; N, 8.40.

### 3.4. Synthesis of **Cav-3-Qx**

A total of 180 mg (0.136 mmol) of **Cav-4-Qx** and 411 mg (0.907 mmol) of CsF were mixed in 48 mL of DMF dry. The reaction was heated at 80 °C, and 15.4 mg (0.136 mmol) of catechol were added. The reaction was quenched by addition of 250 mL of brine. The crude solid was washed with water and purified by column chromatography (CH_2_Cl_2_:EtOAc 95:5) leading to 91.2 mg of **Cav-3-Qx** (60%). ^1^H NMR (CDCl_3_, 500 MHz): δ 8.23 (s, 2H, ArH), 7.92 (dd, *J* = 1.0 Hz, 8.0 Hz, 2H, ArH), 7.86 (bs, 2H, OH), 7.81 (m, 2H, ArH), 7.66 (dd, *J* = 1.0 Hz, 8 Hz, 2H, ArH), 7.54 (t, *J* = 7.0 Hz, 2H, ArH), 7.49–7.43 (m, 4H, ArH), 7.28 (s, 2H, ArH), 7.13 (s, 2H, ArH), 7.08 (s, 2H, ArH), 5.58 (t, *J* = 8.0 Hz, 1H, CH), 5.50 (t, *J* = 8.0 Hz, 2H, CH), 4.25 (t, *J* = 8.0 Hz, 1H, CH), 2.29–2.16 (m, 8H, CH_2_(CH_2_)_3_CH_3_), 1.46–1.24 (m, 32H, CH_2_(CH_2_)_3_CH_3_), 0.93 (t, *J* = 7.0 Hz, 12H, CH_3_). ESI-MS *m/z* 1202 [M+H]^+^. Anal. Calcd. For C_76_H_78_N_6_O_8_ C, 75.85; H, 6.53; N, 6.98; O, 10.64. Found C, 75.77; H, 6.45; N, 6.89. 

### 3.5. Procedure for Fluorescence Titrations

Two mother solutions of host and guest (1.0 × 10^−3^ M) in dry solvent (cavitands were solubilized in CHCl_3_ and DA in EtOH) were prepared. From these, different solutions with different ratio receptor/guest were prepared.

Fluorescence titration of **Cav-4-Qx** and DA was carried out using λ_ex_ = 320 nm in dry CHCl_3_, recording at λ_em_ = 408 nm at 25 °C. Fluorescence titration of **Cav-3-Qx** and DA was carried out in dry CHCl_3_, using λ_ex_ = 340, recording at λ_em_ = 436 nm, at 25 °C. With this data treatment, the apparent binding affinities of receptors with DA were estimated using HypSpec (version 1.1.33, Protonic Software, Florence, Italy)) [54,55,56,57], a software designed to extract equilibrium constants from potentiometric and/or spectrophotometric titration data. HypSpec starts with an assumed complex formation scheme and uses a least-squares approach to derive the spectra of the complexes and the stability constants. Χ^2^ test (chi-square) was applied, where the residuals follow a normal distribution (for a distribution approximately normal, the χ^2^ test value is around 12 or less). In all of the cases, χ^2^ ≤ 10 were found, as obtained by 3 independent measurements sets.

### 3.6. Determination of Stoichiometry

Stoichiometry of the complexes were investigated by the Job’s plot method, using spectrophotometric measurements. The samples were prepared by mixing equimolecular stock solutions (1.0 × 10^−3^ M) of the appropriate host and guest to cover the whole range of molar fractions, keeping constant the total concentration (1 × 10^−5^ M). The changes in absorbance compared to uncomplexed receptor species (ΔA × χ^−1^) were calculated and reported versus the receptor mole fraction (χ). These plots show invariably a maximum at 0.5 mol fraction of receptor, thus suggesting its 1:1 complex formation.

### 3.7. Preparation of Solid Support

The siloxane-based polymeric support was prepared by mixing a precursor based on dodecamethylcyclohexasiloxane with a cross-linking agent (5 wt%) according to the producer instruction. The product is commercially available from Reschimica S.r.l. (Florence, Italy). It was used as received. Following vigorous mixing for about 10 min of the two components, the product was poured onto a solid glass substrate and incubated in an oven at 120 °C for 2 h. The mixture also contains silica to make the final polymer white in color. 

### 3.8. Procedure for Sensing by Strip Test

In a 1.5 × 1.0 cm of siloxane-based polymeric support we dropped on different positions 2 μL of the **Cav-4-Qx** (1 × 10^−3^ M in CHCl_3_) and 2 μL of the phenanthrene (1 × 10^−3^ M in CHCl_3_). The solid sensor was illuminated with a UV lamp (365 nm) in a dark chamber and the visible emission image was acquired with a smartphone (iPhone 13, 24 Mpixel). Then, 10 nebulizations (having a total amount of 460 μL) of a solution of the DA (from 10^−3^ M to 10^−12^ M in EtOH) was nebulized by a conical nebulizer onto the sensor. The amounts of nebulized DA (87 μg~87 fg) have been calculated from the respective concentrations of the solutions of DA and the volume nebulized. After evaporation on air at room temperature of the nebulized solvent, the solid sensor was further photographed, and the images before and after nebulization have been elaborated by Fiji [58]. In particular, images have been converted in RGB channel values, and converted into Gray scale value (G) by using the formula G = (R_value_ + G_value_ + B_value_)/3, thus obtaining a single value for each pixel. The emission intensities of this scale for each probe have been compared to the control (phenanthrene), and these normalized values (ratio between the intensity of the **Cav-4-Qx** probe and the intensity of the control) have been reported. The resulting values were tabulated for statistical treatment using the Excel software (Microsoft 365).

### 3.9. Procedure for Sensing by Strip Test-Experimental Setup

**Figure 6:**
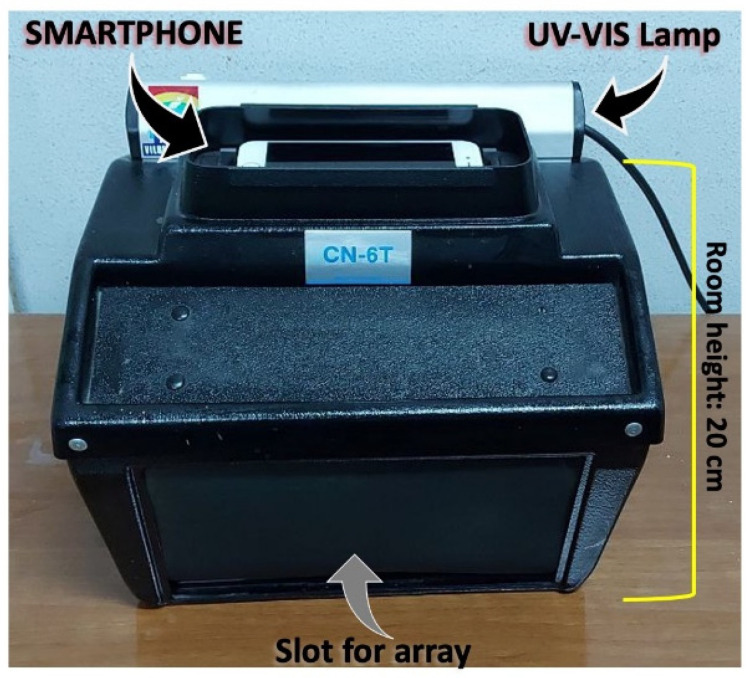
Dark chamber used in this work.

UV-Vis lamp power 6W, excitation wavelength 365 nm. The position of the solid sensor into the dark chamber can be modified, due to the presence of the control probe. In fact, the possible variations of the irradiation are normalized by the comparison with the control. The solid sensor is located at 20 cm from the smartphone and UV source. The dark chamber used is reported in Figure 6.

### 3.10. Recovery

The recovery of the solid sensor was tested by performing an acid–base cycle. Firstly, DA (1.0 × 10^−3^ M in EtOH) was nebulized onto the solid sensor, which was dried in air for 30 s and images have been acquired as reported above. Then, the solid sensor was immerged in a solution of HCl (1.0 × 10^−3^ M in water) for 1 min, in pure water for other 30 s, in a solution of NaOH (1.0 × 10^−3^ M in water) for 1 min, and then in pure water for other 30 s. At the end, the image was acquired. The cycle was repeated three times.

## 4. Conclusions

In conclusion, the possibility to use quinoxaline cavitands for the supramolecular recognition of dopamine has been demonstrated both in solution and in solid state. In fact, the formation of supramolecular complexes with dopamine has been supported by fluorescence titrations, ESI-MS and ROESY measurements, with a nanomolar detection limit in solution. In addition, the tetraquinoxaline cavitand has been used to create a solid sensor by using siloxane-based polymeric as solid support. This new device shows a linear emission response to DA in the range 10 mM~100 pM, with a 1 pM detection limit on solid phase. Due to the DA concentration values in the common human fluids (0.13 mM in plasma, 7 mM in urine and 0.124 nM in saliva), this study represents *proof of concept* for the realization of real sensors for the easily detection of human dopamine. Further studies are ongoing to (i) optimize the deposition of the cavitand onto the siloxane-based polymeric support (also by an inkjet-printer); (ii) optimize the portability of the system, by using commercial UV lamps and optical fibers as detector, and (iii) detect other important biomarkers, such as cortisol, by using the array technology.

## Data Availability

**Cav-4-Qx**, **Cav-3-Qx** and all data reported in this work can be obtained by the authors, after formal request.

## Supplementary Materials

The following supporting information can be downloaded at: https://www.mdpi.com/article/10.3390/molecules27217503/s1, Fluorescence titrations, Job’s Plots, HypSpec results, ESI-MS of supramolecular complexes, ROESY spectra.

## Sample Availability

Samples of the compounds **1**, **Cav-4-Qx** and **Cav-3-Qx**, are available from the authors.
