# Supplementary material for: Smartphone-Based Dopamine Detection by Fluorescent Supramolecular Sensor
Source: Molecules. 2022 Nov 3;27(21):7503. doi: 10.3390/molecules27217503 (PMC9654496; doi:10.3390/molecules27217503)
Supplement: Supplementary file 1 [file molecules-27-07503-s001.zip › molecules-1986236-supplementary.pdf]

SMARTPHONE-BASED DOPAMINE DETECTION BY FLUORESCENT SUPRAMOLECULAR SENSOR

Rossella Santonocito, Nunzio Tuccitto, Andrea Pappalardo and Giuseppe Trusso Sfrazzetto*

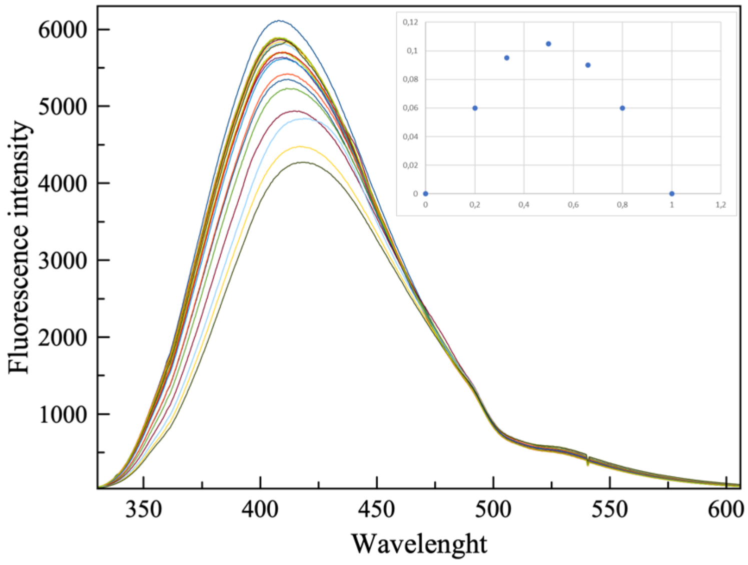

Figure S1. Fluorescence titration between **Cav-4-Qx** and DA ($CHCl_3$, [**Cav-4-Qx**] = 1 x 10^{-5} M, DA additions were in the 0-11 equivalent range). Inset shows the relative Job's Plot.

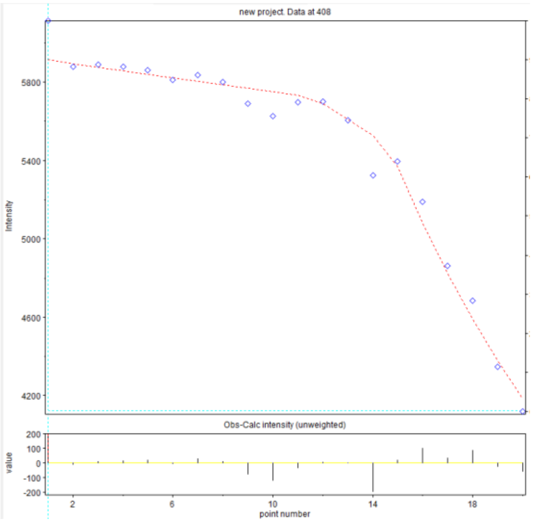

		standard
Log beta	value	deviation
AB	3.332	0.0286

Figure S2. HypSpec Plot and relative output file

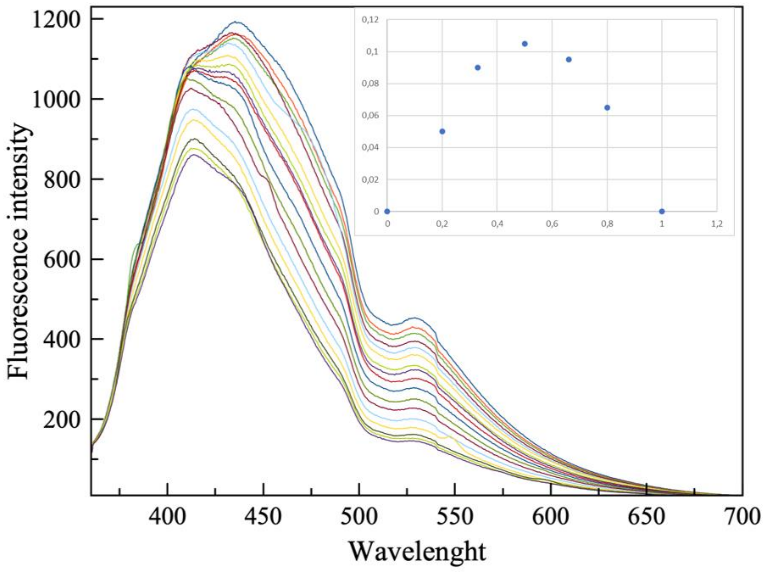

Figure S3. Fluorescence titration between **Cav-3-Qx** and DA ($CHCl_3$, [**Cav-3-Qx**] = 1 x 10^{-5} M, DA additions were in the 0-11 equivalent range). Inset shows the relative Job's Plot.

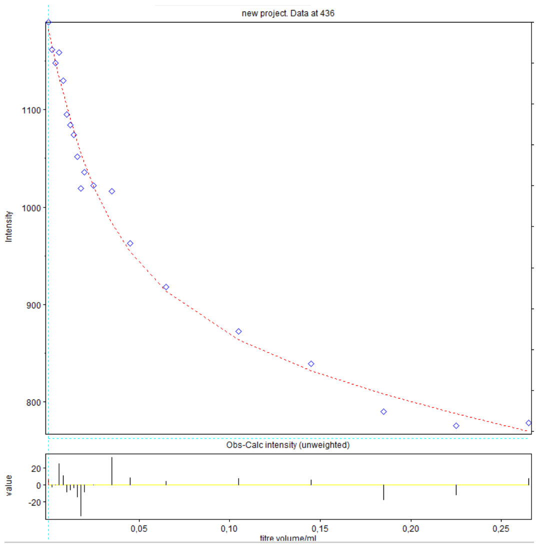

Log beta	value	standard deviation
AB	4.9821	0.0857

Figure S4. HypSpec Plot and relative output file

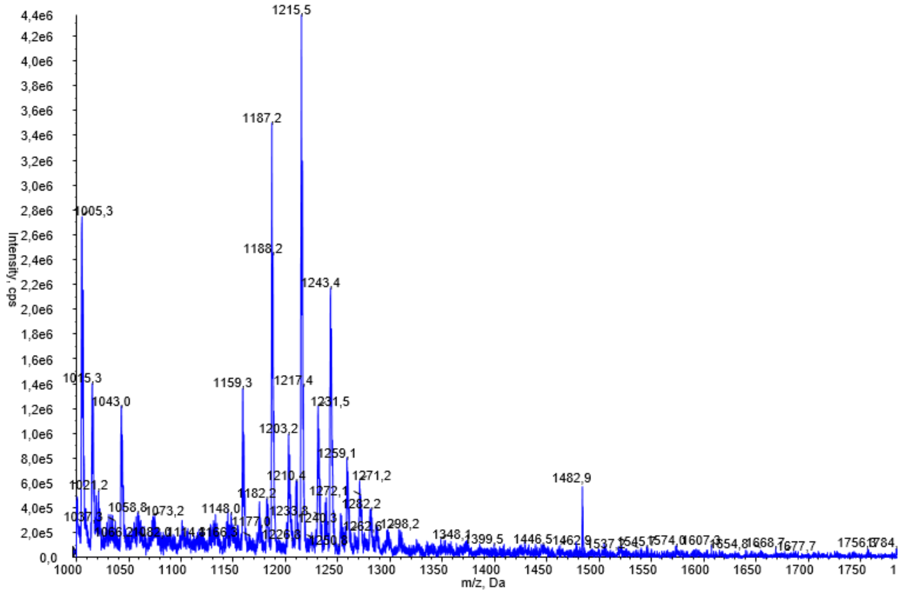

Figure S5. ESI-MS spectrum of **Cav-4-Qx** with 1 equivalent of DA in CH_3CN.

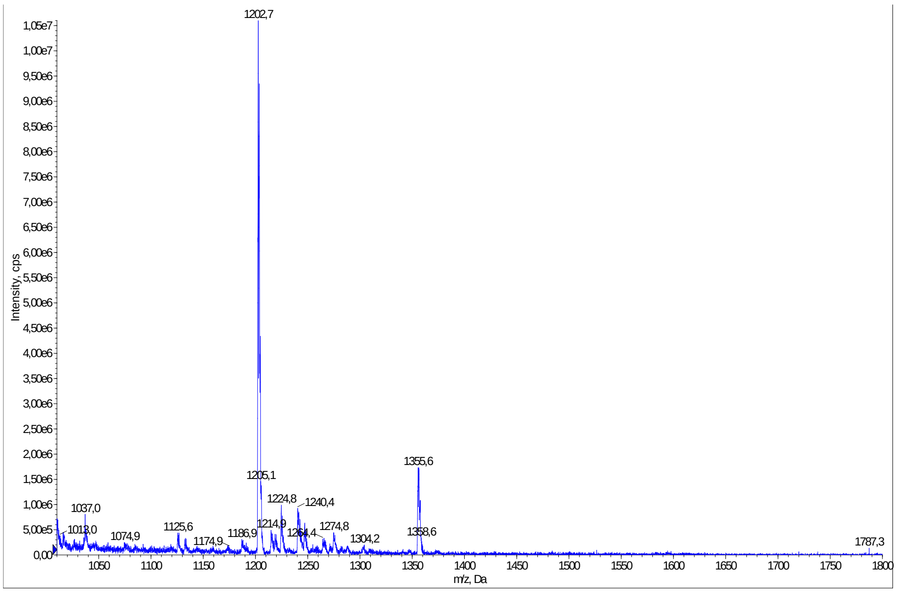

Figure S6. ESI-MS spectrum of **Cav-3-Qx** with 1 equivalent of DA in CH_3CN.

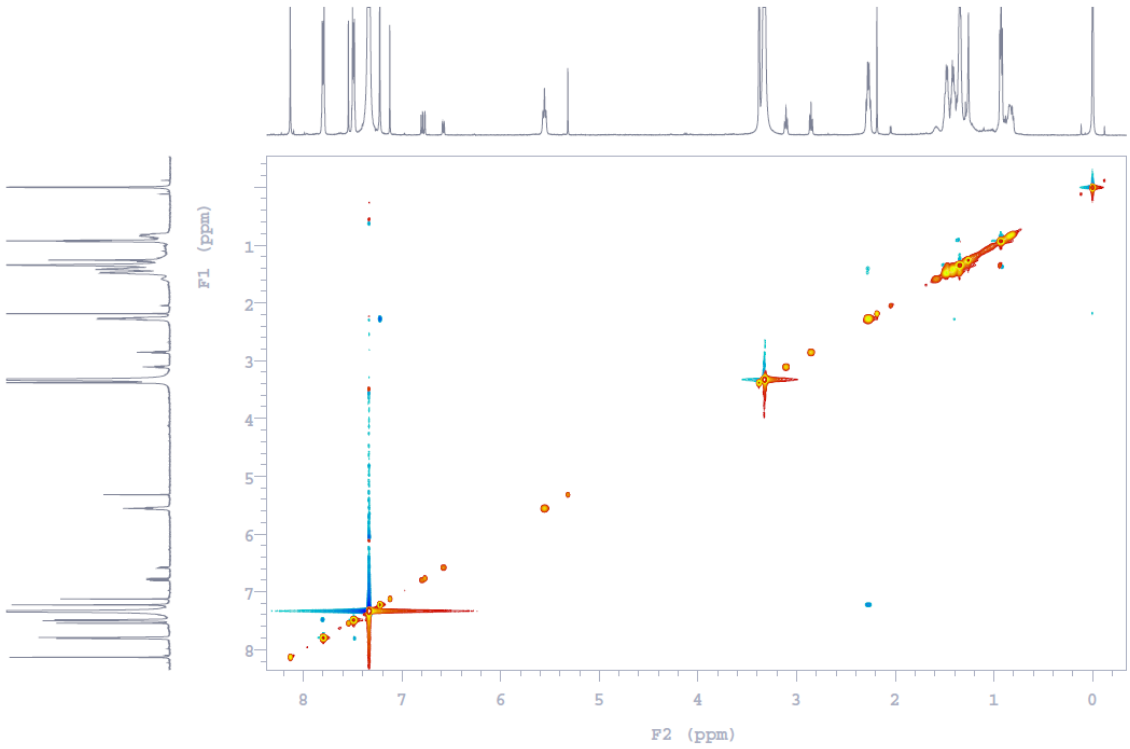

Figure S7. ROESY spectrum of **Cav-4-Qx** (1×10^{-3} M, $CDCl_3/CD_3OD$: 6/1) with 1 equivalent of DA.

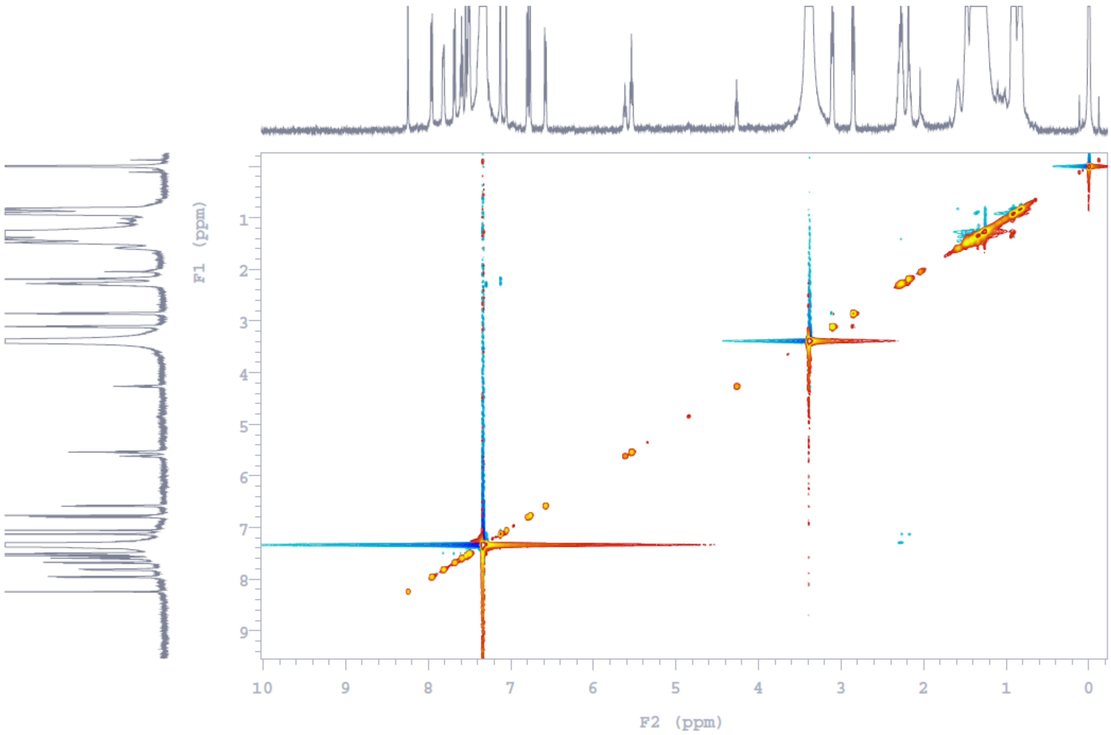

Figure S8. ROESY spectrum of **Cav-3-Qx** (1×10^{-3} M, $CDCl_3/CD_3OD$: 6/1) with 1 equivalent of DA.
